# Microfibrillated Cellulose Grafted with Metacrylic Acid as a Modifier in Poly(3-hydroxybutyrate)

**DOI:** 10.3390/polym13223970

**Published:** 2021-11-17

**Authors:** Marius Stelian Popa, Adriana Nicoleta Frone, Ionut Cristian Radu, Paul Octavian Stanescu, Roxana Truşcă, Valentin Rădiţoiu, Cristian Andi Nicolae, Augusta Raluca Gabor, Denis Mihaela Panaitescu

**Affiliations:** 1 Institute for Research & Development in Chemistry and Petrochemistry—ICECHIM, 202 SplaiulIndependentei, 060021 Bucharest, Romania; popamarius7777@gmail.com (M.S.P.); adriana.frone@icechim.ro (A.N.F.); vraditoiu@icechim.ro (V.R.); cristian.nicolae@icechim.ro (C.A.N.); raluca.gabor@icechim.ro (A.R.G.); 2 Faculty of Applied Chemistry and Materials Science, University Politehnica of Bucharest, 1–7 Gh. Polizu Street, 011061 Bucharest, Romania; ionut_cristian.radu@upb.ro (I.C.R.); paul.stanescu@upb.ro (P.O.S.); 3 National Research Centre for Micro and Nanomaterials, University Politehnica of Bucharest, 313 Spl. Indendentei, 060042 Bucharest, Romania; truscaroxana@yahoo.com

**Keywords:** microfibrillated cellulose, polymethacrylic acid, grafting, poly(3-hydroxybutyrate), biocomposites, compatibility

## Abstract

This work proposes a new method for obtaining poly(3-hydroxybutyrate) (PHB)/microfibrillated cellulose (MC) composites with more balanced properties intended for the substitution of petroleum-based polymers in packaging and engineering applications. To achieve this, the MC surface was adjusted by a new chemical route to enhance its compatibility with the PHB matrix: (i) creating active sites on the surface of MC with γ-methacryloxypropyltrimethoxysilane (SIMA) or vinyltriethoxysilane (SIV), followed by (ii) the graft polymerization of methacrylic acid (MA). The high efficiency of the SIMA-MA treatment and the lower efficiency in the case of SIV-MA were proven by the changes observed in the Fourier transform infrared FTIR spectra of celluloses. All modified celluloses and the PHB composites containing them showed good thermal stability close to the processing temperature of PHB. SIMA-modified celluloses acted as nucleating agents in PHB, increasing its crystallinity and favoring the formation of smaller spherulites. A uniform dispersion of SIMA-modified celluloses in PHB as a result of the good compatibility between the two phases was observed by scanning electron microscopy and many agglomerations of fibers in the composite with unmodified MC. The dual role of SIMA-MA treatment, as both compatibilizer and plasticizer, was pointed out by mechanical and rheological measurements. This new method to modify MC and obtain PHB/MC composites with more balanced stiffness–toughness properties could be a solution to the high brittleness and poor processability of PHB-based materials.

## 1. Introduction

The poor disposal of waste combined with the wasteful mentality of humankind has led to well-known environmental problems. Currently, petroleum is used in everything that requires electricity [1], and the dependence on petroleum-based plastics increases the demand for oil, which drags along an increased need for energy, and so on. The focus of research in recent years has been to reduce this demand by exploring more natural-based materials with the aim to replace classic, fossil-based plastics [2,3,4]. Promising results have been obtained with poly-(3-hydroxybutyrate) (PHB), an aliphatic microbial polyester that shows similar mechanical and thermal properties to polypropylene with the added bonuses of better barrier properties and biodegradability [5]. It is biosynthesized by certain bacteria as a means of stocking energy in nitrogen- and phosphorus-deficient conditions [6]. However, in its pure form, PHB is highly brittle at room temperature and has a low processing window, which limits its possibilities of application. Thus, different approaches such as plasticization, melt blending with other polymers, copolymerization, and preparation of nanocomposites have been studied to overcome these drawbacks [7]. The introduction of organic or inorganic fillers has proven to be an attractive and versatile technique to enhance the performances of PHB. Several reinforcing agents have been incorporated into PHB for improving its properties, such as (nano)clays [8], carbon nanotubes [9], graphene [10], and cellulosic materials [11].

Cellulose is one of the most abundant and studied materials in the world and can be obtained in various sizes and shapes [12]. It can be extracted from a variety of sources, such as wood and plants, and can be biosynthesized by some bacterial strands as extracellular material [13]. Its versatility regarding geometry, from (nano)fibers to (nano)particles, and its sources allows cellulose to be used in countless applications. Cellulose micro- and nanofillers have been used to improve the thermal and mechanical properties of PHB [11,14,15]. A big issue in the case of PHB–cellulose composites is the poor compatibility between the matrix and the filler due to the hydrophobic nature of PHB and the strong hydrophilicity of cellulosic fillers [16]. The methods attempted so far to improve this compatibility, such as the treatment of cellulose fillers by acetylation, silylation, and TEMPO-mediated or plasma oxidation, have led to some improvement in PHB properties [17,18,19]. Reactive extrusion has also been used to improve the adhesion at the PHB–cellulose interface, leading to a moderate enhancement of mechanical properties [20].

A different route for improving the compatibility between PHB and microfibrillated cellulose was attempted in this paper. The hypothesis underpinning this work is that cellulose modification with polymethacrylic acid units improves its compatibility with PHB. Indeed, only a limited number of polymers are miscible with PHB, such as polyvinyl acetate or polymethyl acrylate [21,22]. An et al. have shown that polymethyl acrylate is fully miscible with PHB over the entire composition range [22]. This may be presumed from the similarity of the repeating units, both vinyl acetate and methyl acrylate units being isomers of 3-hydroxybutyrate (HB). Another isomer of the HB unit is methacrylic acid, which is used in this work to modify microfibrillated cellulose. Thus, polymethacrylic acid (PMA) grafts were grown on the surface of cellulose to increase the compatibility between PHB and the cellulosic filler. Although polymethacrylic acid was previously grafted on a few cellulosic substrates [23,24], especially for medical application, no attempt to modify cellulose with PMA for increased compatibility with PHB has been performed so far. In addition, this is the first attempt to pre-activate the cellulose surface with various silanes for better grafting of PMA and obtaining improved properties. In this work, Fourier transform infrared spectroscopy (FTIR) and thermogravimetric analysis (TGA) were used to highlight the grafting of silanes and the presence of polymethacrylic acid on the surface of cellulose. The influence of modified celluloses on the morphology, thermal, and mechanical properties of PHB composites was also studied.

## 2. Experimental Section

### 2.1. Materials

Microcrystalline cellulose (MCC) with an average diameter of 20 µm and purchased from Sigma Aldrich (Saint Louis, MO, USA) was used to obtain the microfibrillated cellulose (MC). Methacrylic acid (MA, purity 99%), vinyltriethoxysilane (SIV) (purity 97%), and 2,2′-azobis(2-methylpropionitrile) (AIBN) (purity > 98%) were purchased from Sigma Aldrich (Saint Louis, MO, USA). Moreover, γ-methacryloxypropyltrimethoxysilane (SIMA) (purity 98%, trade name—Xiameter OFS-6030 silane) was obtained from Dow Corning Co. (Midland, MI, USA) and acetone (purity > 99.92%) was obtained from Chimreactiv (Bucharest, Romania). All chemicals were used without further purification. PHB pellets (P304) from Biomer (Schwalbach am Taunus, Germany) with a density of 1.24 g/cm^3^ were used as the polymer matrix in the composites.

### 2.2. Preparation of Microfibrillated Cellulose

MC was originally obtained from a 2 wt% MCC suspension in distilled water, which was maintained at room temperature for 48 h to enable MCC soaking. Then, the MCC suspension was processed by a high-pressure mechanical treatment for 12 cycles at 200 MPa using a microfluidizer LM20 (Microfluidics, Westwood, MA, US). A cellulose gel resulted from the treatment. This was first frozen at −20 °C for 48 h and then freeze-dried (FreeZone 2.5 L, Labconco, Kansas-City, MO, USA) at −85 °C and 0.006 mbar for 48 h. The dried cellulose was further milled using an ultra-centrifugal mill ZM 200 (Retsch GmbH & Co., Düsseldorf, Germany) at a speed of 6000 min^−1^, resulting in microfibrillated cellulose (MC) powder.

### 2.3. Silanization Reaction

**Figure 1:**
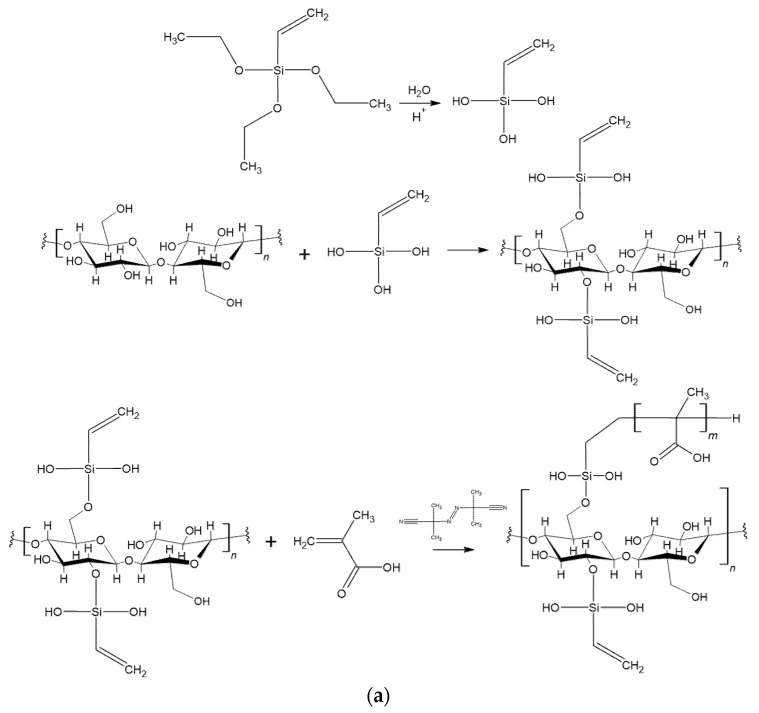

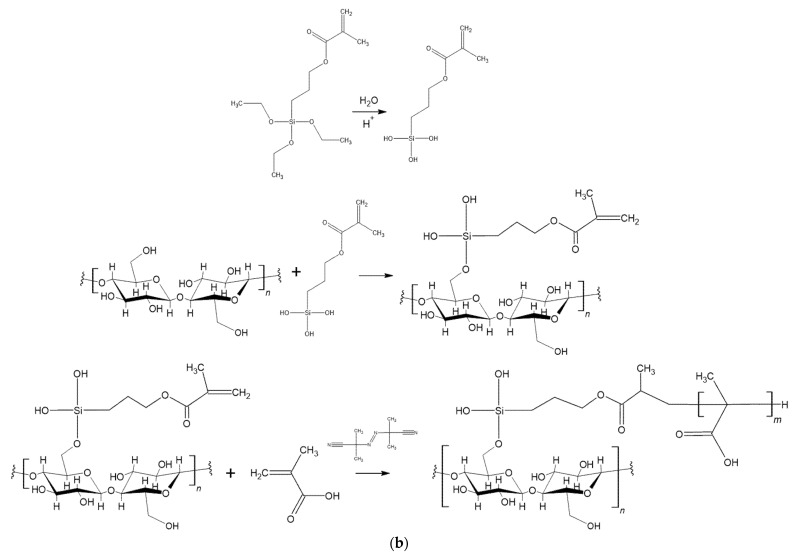
Reaction schemes for the chemical modification of microfibrillated cellulose for obtaining: (**a**) MC-SIV and MC-SIV-MA, (**b**) MC-SIMA and MC-SIMA-MA.

MC powder was soaked in water (10 wt%) under magnetic stirring for 8 h at room temperature. A silane solution (5 wt%) was prepared by dissolving either SIV or SIMA in water at room temperature under strong stirring for 30 min. The silane solution was acidified with glacial acetic acid until the pH value of 3.5 and allowed to hydrolyze for 30 min. Then, the hydrolyzed silane solution was poured over the MC suspension and allowed to react at 50 °C under strong magnetic stirring for another 4 h. Following this, the modified celluloses were washed three times with a plentiful amount of distilled water. The suspensions were then freeze-dried and the dried celluloses were milled using the same equipment and conditions as previously mentioned, resulting in MC-SIV or MC-SIMA. The reaction schemes for the chemical modification of cellulose by silanization with SIMA and SIV are shown in Figure 1.

### 2.4. Polymerization Reaction

MC-SIV and MC-SIMA powders were soaked in acetone, an aprotic solvent that was chosen as reaction medium. In the meantime, methacrylic acid (MA) was dissolved in acetone at room temperature left under magnetic stirring for 8 h. Trace amounts of acetone were used to dissolve the initiator, AIBN. Overall, the methacrylic acid/cellulose ratio was 1/2 *v*/*w*, and the AIBN concentration was 10^−2^ mol/L regarding MA volume. Next, all components were mixed together and allowed to react under reflux for 4 h. The modified celluloses were washed with distilled water, freeze-dried, and milled, similar to the previous step. Polymethacrylic acid-modified celluloses, MC-SIV-MA and MC-SIMA-MA, were thus obtained. Figure 1 presents the reaction schemes for the MA graft polymerization on the surface of silanized celluloses.

### 2.5. Preparation of the PHB/Modified Cellulose Composite Films

The composite films were prepared by mixing PHB and modified celluloses (2 wt%) in a 30 cm^3^ Brabender mixing chamber(Brabender GmbH & Co. KG, Duisburg, Germany) at 165 °C for 7 min, followed by molding on a two-rollmill to obtain sheets. These were further compression-molded in a P200E press (Dr. Collin, Ebersberg, Germany) at 175 °C with 120 s of preheating at 0.5 MPa and 60 s of compression at 10 MPa. A cooling cassette accessory was used for the rapid cooling of the films.

### 2.6. Characterization

#### 2.6.1. Fourier Transform Infrared Spectroscopy

The FTIR attenuated total reflectance (ATR) analysis was carried out on a JASCO 6300 spectrophotometer (JASCO International Co., Ltd., Tokyo, Japan) equipped with a Specac ATR Golden Gate unit (Specac, Inc., Orpington, UK) with KRS5 lens. FTIR spectra were scanned from 4000 to 400 cm^−1^, with 32 scans per spectrum at a resolution of 4 cm^−1^.

#### 2.6.2. Thermogravimetric Analysis

Original and differently modified MCs and the composites containing these fibers were characterized by TGA using a TA-Q5000 (TA Instruments, New Castle, DE, USA). Measurements were carried out on samples of 8–10 mg from room temperature to 700 °C with 10 °C/min in nitrogen atmosphere purged with 40 mL/min.

#### 2.6.3. Differential Scanning Calorimetry (DSC)

Calorimetric measurements were carried out on a DSC Q2000 from TA Instruments (New Castle, DE, USA) under a helium flow of 25 mL/min using 10–13 mg from each sample and a heating/cooling rate of 10 °C/min during cycles. The applied program involved: a rapid cooling from the ambient temperature to −60 °C, equilibration for 2 min at this temperature, heating to 200 °C (first heating cycle), equilibration for 2 min, cooling to −60 °C (cooling cycle), equilibration for 2 min and heating again to 200 °C (second heating cycle). The crystallinity (*X_c_*) was calculated from the second heating cycle with:

(1) $$X_{c}\;(\%)\;\;=\frac{\Delta H}{\Delta H_{0}\;\times \;w_{PHB}}\;\times \;100$$

The formula includes the melting enthalpy (∆H) and the weight fraction (w_PHB_) of PHB in the composite along with the melting enthalpy of 100% crystalline PHB (ΔH_0_ = 146 J/g [25]).

#### 2.6.4. Dynamic Mechanical Analysis (DMA)

Composite specimens with the length × width × thickness of 12.7 mm × 6.8 mm × 0.8 mm were cut from the compression-molded films and analyzed with a DMA Q800 (TA Instruments, New Castle, DE, USA) in multifrequency-strain mode using a tension clamp. The specimens were cooled from the ambient temperature to −50 °C with 10 °C/min, equilibrated for 2 min at this temperature, and heated to 125 °C with a heating rate of 3 °C/min.

#### 2.6.5. Tensile Properties

The tensile properties of PHB compression-molded films were determined according to ISO 527 using an Instron 3382 universal testing machine (Instron, Norwood, MA, USA) with a 10 kN load cell. Five specimens were tested for each sample at room temperature with a crosshead speed of 10 mm/min. The average mechanical properties, Young’s modulus, and tensile strength and elongation at break were calculated as mean and standard deviation using the Bluehill 2 software.

#### 2.6.6. Scanning Electron Microscopy (SEM)

Morphological aspects of composites in the fracture were investigated with a Quanta Inspect F Scanning Electron Microscope (Philips/FEI, Eindhoven, The Netherlands), with a resolution of 1.2 nm at an accelerating voltage of 30 kV. Prior to the measurements, the samples were frozen in liquid nitrogen and fractured, then the fractures were sputter-coated with gold for a better contrast. 

The MC powder obtained after freeze-drying and milling was analyzed with the same scanning electron microscope. Before the measurements, the powder was spread on an adhesive tape and sputter-coated with gold.

#### 2.6.7. Polarized Light Optical Microscopy

Polarized light optical microscopy (POM) analysis was carried out using an Olympus BX53F Microscope equipped with a DP23/DP28 Digital Camera (Olympus, Tokyo, Japan). Composite films of 20–30 μm in thickness were used for the measurements. The films were obtained by compression molding at 175°C (preheating for 150 s and compression for 65 s at 10 MPa). Sections of the films were melted between glass slides in an oven at 220 °C for 5 min, kept at room temperature for about 15 min, and then analyzed by POM.

## 3. Results and Discussion

### 3.1. Morphological Investigation of MC Powder

**Figure 2:**
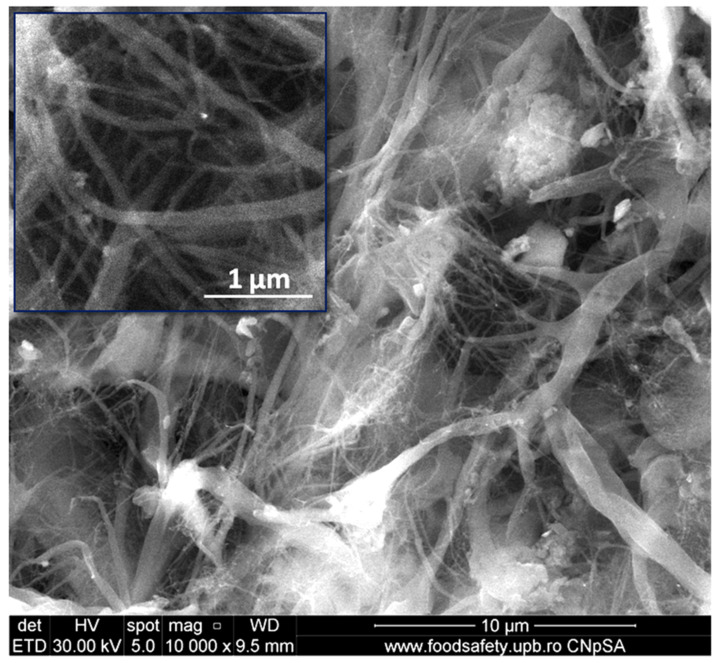
SEM image of microfibrillated cellulose after freeze-drying and milling.

Figure 2 shows the SEM image of dried microfibrillated cellulose powder. One may observe a network of micro- and nanofibers. Most of the nanofibers had a width of less than 100 nm and a length of a couple of microns. This is better seen in the detail in the upper-left corner of Figure 2. Microfibers with a width of less than 1 µm may also be observed. It turns out that 12 cycles of high-pressure homogenization were sufficient to defibrillate microcrystalline cellulose with an initial width of about 20 µm in submicron-sized fibers.

Many entangled and agglomerated cellulose nanofibers were noticed in Figure 2. This is an effect of the removal of water during the freeze-drying process that favors the self-aggregation of individual nanofibers. However, the aggregates disentangled during the melt mixing with the polymer. A network of cellulose fibers with similar size was obtained, starting with delignified Fique tow using a TEMPO oxidation process coupled with ultrasonication [26].

### 3.2. Characterization of Modified Celluloses

#### 3.2.1. FTIR Analysis

**Figure 3:**
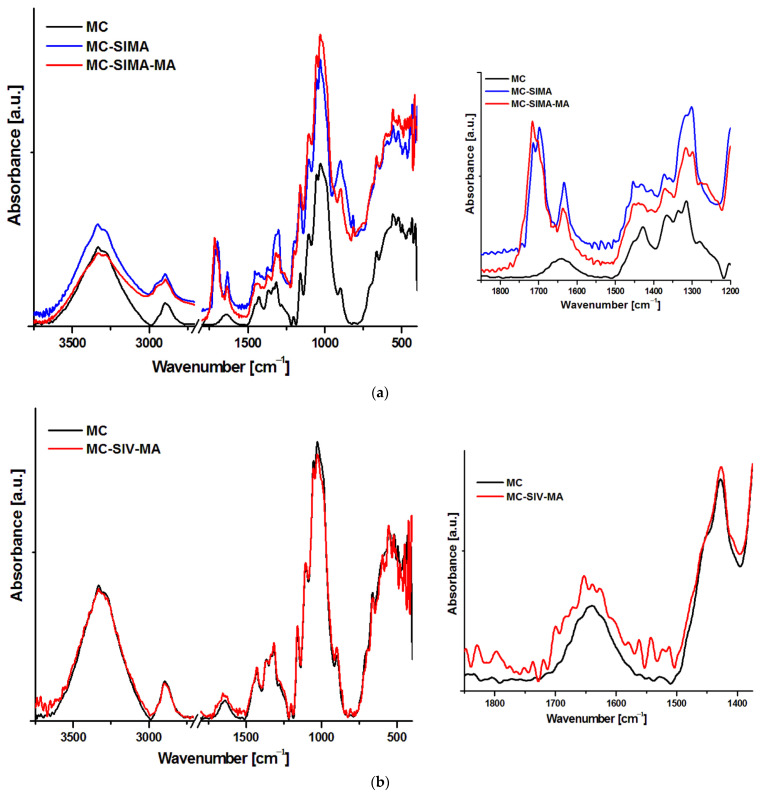
FTIR spectra of MC, MC-SIMA, and MC-SIMA-MA (**a**) and MC-SIV-MA (**b**).

Figure 3 presents the FTIR spectra of the MC and modified celluloses. The unmodified MC spectrogram shows the expected broad peak centered on 3333 cm^−1^, characteristic of the stretching vibration of hydrogen bonded –OH groups of the cellulose, which does not significantly change after chemical reactions.

In unmodified cellulose, the peak from 2895 cm^−1^, which is attributed to C–H stretching vibrations, suffers changes after chemical modifications; thus, a shoulder appears in both SIMA-modified celluloses at about 2950 cm^−1^ (Figure 3a) due to the stretching of CH_2_ groups in the oxypropyl chain of SIMA [27]. Further, the broad small peak at 1640 cm^−1^, attributed to the bending vibration of the –OH groups in water, was overlapped by a new peak located at 1633 cm^−1^ in MC-SIMA and a lower intensity broad peak at 1637/1630 cm^−1^ in MC-SIMA-MA, which may be assigned to the stretching vibrations of unreacted C=C groups of the acrylic moiety [27,28]. New peaks with different shapes and intensities appeared between 1750 and 1650 cm^−1^ in MC-SIMA and MC-SIMA-MA (Figure 3a—detail). Two peaks were observed at 1714 cm^−1^ and 1698 cm^−1^ in MC-SIMA spectrum, deriving from the stretching vibrations of the C=O groups in the methacryloyl chains of the grafted silane [28]. A strong peak at 1716 cm^−1^ and three shoulders at 1701, 1740, and 1690 cm^−1^ were observed in the case of MC-SIMA-MA (Figure 3a—detail). As specified above, the peaks at 1716 and 1701 cm^−1^ may be ascribed to the carbonyl groups of silane, however, the shoulder at 1690 cm^−1^ can be attributed to the stretching vibration of the C=O groups in the poly(methacrylic acid) [29]. The shoulder from 1740 cm^−1^ belonged to the ester carbonyl signal and shows the formation of the cellulose ester in the case of MC-SIMA-MA [30]. Therefore, the acylation of cellulose was proved by the appearance of the new signal at 1740 cm^−1^ along with the decreased intensity of the band that is characteristic of the hydrogen bonded −OH in MC-SIMA-MA from 3333 cm^−1^. However, the weak signal of the ester C=O group shows that this reaction was only a secondary one.

In the case of MC modified by vinyl silane and methacrylic acid, the changes observed in the FTIR spectra (Figure 3b) were minor. The small changes noticed in the band from 1600 to 1700 cm^−1^ (Figure 3b—detail) may have derived from the stretching vibration of the C=O groups in the poly(methacrylic acid) (1696 cm^−1^) and the C=C stretching in the terminal vinyl groups of methacrylic acid oligomers, or in the unreacted vinyl silane (1637 cm^−1^). However, the signals were so weak that it can therefore be presumed that the chemical modification of cellulose was less efficient in this case.

#### 3.2.2. Thermogravimetric Analysis of Modified Celluloses

**Figure 4:**
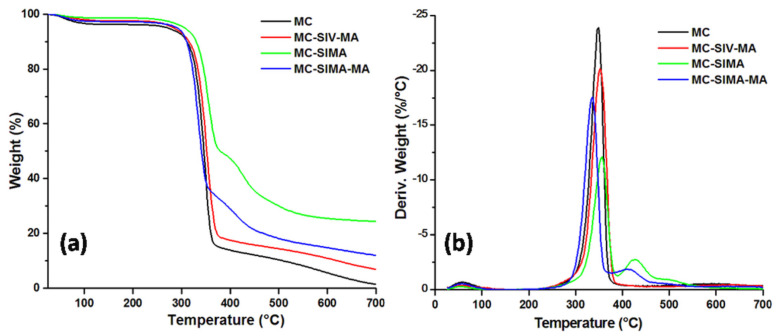
Thermogravimetric (**a**) and derivative (**b**) curves of MC and modified celluloses.

Figure 4 shows the thermograms of the MC and the modified celluloses. After the loss of water at up to 100 °C, MC decomposed between 270 and 370 °C when it lost 80% of its weight. A significantly increased thermal stability was observed after the treatment of microfibrillated cellulose with SIMA. Thus, the temperature at 5% weight loss (T_5%_) increased by about 34 °C after SIMA treatment. Further grafting with MA led to an increase in T_5%_ by 20 °C for MC-SIMA-MA and by 14 °C for MC-SIV-MA (Table 1). Several differences in the degradation process were noticed depending on the treatment. Thus, the temperature at the maximum decomposition rate (T_max_) increased in the case of MC-SIMA by 8 °C and for MC-SIV-MA by about 5 °C, and decreased for MC-SIMA-MA by 13 °C (Table 1). The increased thermal stability after the treatment of MC with silanes was an effect of the oligomers formed by the condensation of silanes at increased temperature that act as a protective barrier and delay the degradation of cellulose, similar to other observations [31,32]. Indeed, a new peak was observed after that characteristic to the decomposition of cellulose in the derivative curves of MC-SIMA (427 °C) and MC-SIMA-MA (415 °C), which probably derived from the thermal decomposition of siloxanes [33]. The lower T_max_ of MC-SIMA-MA may have been caused by the breaking of the esteric bonds in grafted SIMA or of the cellulose–silane bonds in the presence of the initiator (Figure 1b), leading to the removal of the silane and the slightly lower thermal stability of cellulose.

The residue at 700 °C wasvery high in MC-SIMA (24%) due to the presence of the crosslinked polysiloxanes and was proof of the successful silanization of cellulose. The halved residue obtained in MC-SIMA-MA supports the hypothesis of cellulose–silane bonds breaking and the removal of the silane, also highlighted by the lower T_max_ of MC-SIMA-MA. The lower weight loss at 200 °C (WL_200°C_) of modified celluloses shows their good thermal stability close to the processing temperature of PHB composites.

### 3.3. Characterization of Composites

#### 3.3.1. TGA Analysis

**Figure 5:**
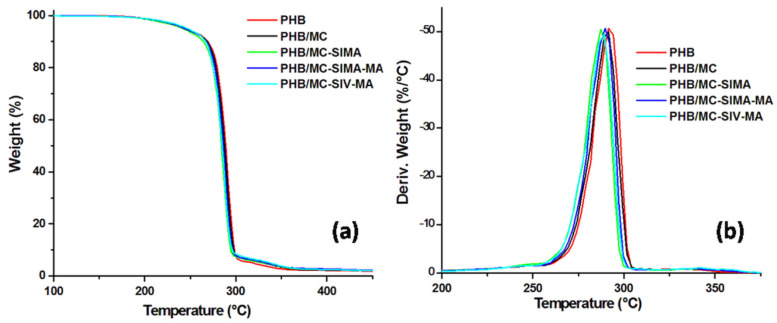
TGA (**a**) and DTG (**b**) curves of composites with differently modified celluloses.

The TGA and DTG curves of PHB composites with 2 wt% modified celluloses are shown in Figure 5. The incorporation of MC and modified celluloses had a small influence on the thermal stability of PHB. The main decomposition step occurred between 250 and 300 °C in neat PHB and in all the composites. The T_5%_ and T_max_ of PHB slightly decreased by less than 5 °C after the addition of celluloses. A similar decrease inthe thermal stability of PHB was reported after the addition of 2 wt% nanofibrillated bacterial cellulose [15], and a stronger decrease was noticedin the case of poly(3-hydroxybutyrate-co-3-hydroxyvalerate) composites with 2.5–10 wt% nanofibrillated cellulose [34]. The greatest influence on the thermal stability of PHB was noticed in the case of MC-SIMA, the cellulose which had undergone the strongest modification, as demonstrated by FTIR and TGA (Figure 3 and Figure 4). Indeed, the esteric bond of SIMA grafted on MC was labile enough to undergo breaking at a high processing temperature (165 °C), releasing free methacryloyl radicals, which can promote the thermal degradation of PHB [35]. However, these processes have a low intensity and their contribution to the PHB degradation is minor, as proven by the small changes in the T_5%_ and T_max_ (Table 2). Similarly, the residue at 700 °C of PHB shows little variation after the addition of modified celluloses (Table 2).

#### 3.3.2. Differential Scanning Calorimetry

**Figure 6:**
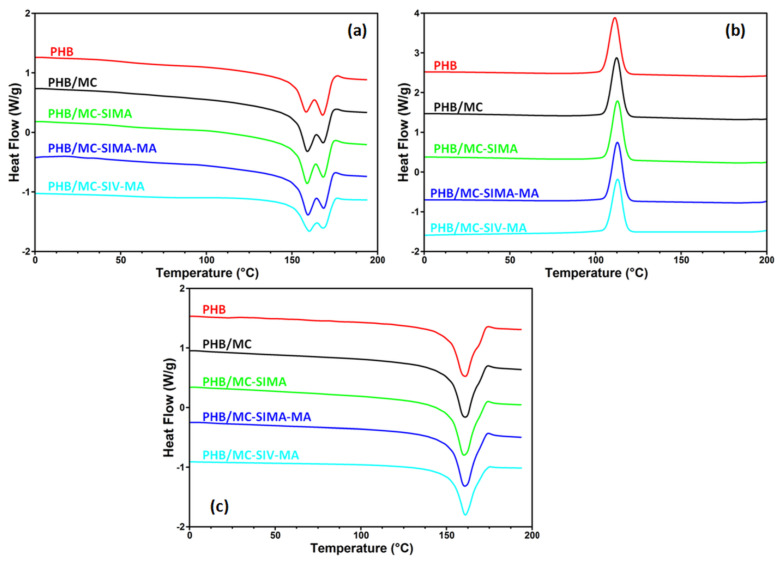
DSC first heating (**a**) and cooling (**b**), and second heating (**c**) of the composites.

Figure 6 presents the behavior of the composites upon heating and cooling, while the crystallinity degree (X_c_), melting (T_m__1_, T_m__2_), and crystallization (T_c_) temperatures along with corresponding enthalpies (ΔH_m1_, ΔH_m2_, ΔH_c_) are listed in Table 3. Double endothermic melting peaks were observed in neat PHB and composites during the first heating cycle (Figure 6a). The phenomenon is generally ascribed to the melt–recrystallization mechanism [11,36]: the first peak arises from the melting of PHB fraction that was formerly crystallized during the compression molding of the films, while the second peak from 168 °C can be related to the melting of the recrystallized PHB fraction during heating. One can observe that the addition of modified celluloses only influenced the peak from the lower temperature (Figure 6a). Thus, a slight shift of this peak to a higher temperature along with an increase in intensity was observed in all the composites. This behavior can be associated with the interactions between the cellulose fibers and PHB, which restrict the flexibility of the polymer chain and increase the melting temperature. The higher intensity of the lower temperature peak in composites shows a higher proportion of smaller and less perfect crystallites, possibly an effect of the nucleating effect of the cellulose fibers. No obvious effect of modified celluloses on the crystallization temperature of the composites was noticed, regardless of the treatment, meaning that all the composites had a crystallization rate similar to that of neat PHB (Figure 6b, Table 3).

A single melting (T_m2_) event was observed in the second heating cycle for all the composites and the small influence of cellulose on it , regardless of the treatment (Figure 6c). However, the degree of crystallinity was higher in composites than in PHB, except for PHB/MC-SIV-MA (Table 3), showing the nucleating effect of MC and modified celluloses, in agreement with previous results [11,34]. The increase in *X_c_* in PHB composites may contribute to an improvement intheir mechanical properties relative to the reference matrix.

The different influence of SIMA- and SIV-modified celluloses upon PHB crystallinity and its thermal behavior can be explained by the different chemical compositions of the two modifiers; methacrylic and polymethacrylic pendant units have better interaction with the PHB matrix as opposed to the less reactive vinyl silane group. In addition, the action of unreacted SIV as a crosslinker in PHB cannot be excluded. This is in agreement with the slightly higher melting temperature and lower crystallinity of the PHB/MC-SIV-MA composite. The crystallization behavior of PHB composites was also investigated by polarized light microscopy (POM), as discussed below.

#### 3.3.3. Polarized Optical Microscopy

**Figure 7:**
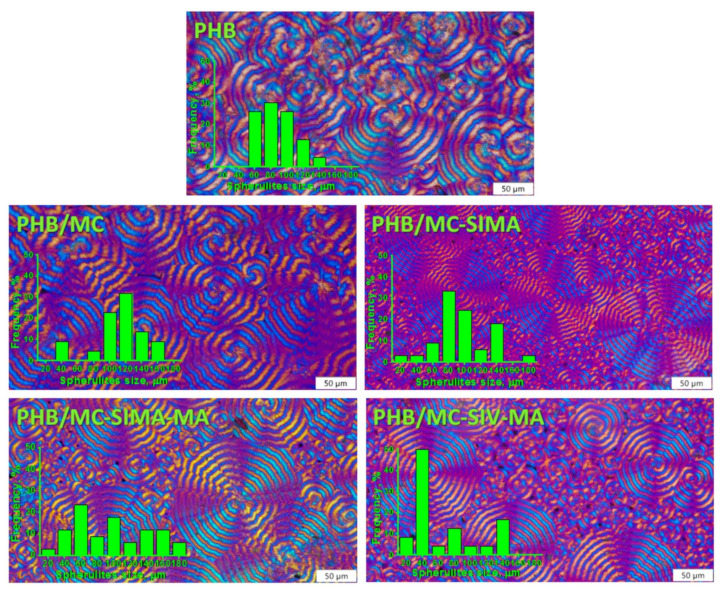
Polarized optical micrographs of PHB and composites with differently modified celluloses (×40); scale bar 50 µm.

The formation of spherulites in PHB and in composites and their shape and size can be observed from the images obtained with the polarized optical microscope presented in Figure 7.

POM images show ring-banded spherulites with the characteristic Maltese cross in both PHB and its composite. In PHB, the spherulites’ size varied between 60 and 140 µm and in composites between 20 and 180 µm, with a greater proportion of smaller spherulites in the composites with treated celluloses, as observed in the histograms attached to the representative POM images (Figure 7). This supports the nucleating effect of celluloses, and of the modified celluloses especially, which is in agreement with the DSC results.

#### 3.3.4. Dynamical Mechanical Analysis

**Figure 8:**
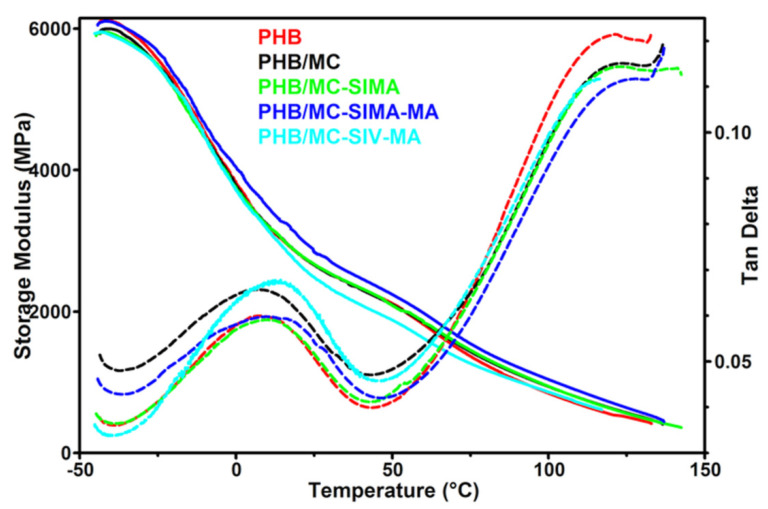
Storage modulus and tan δ of the composites vs. temperature.

The mechanical behavior of the composite films was investigated by DMA and the storage modulus (E’) and tan δ variation with temperature are presented in Figure 8. The glass transition temperature of PHB (T_g_), determined from the tan δ vs. temperature curve, was not changed by the addition of untreated MC but increased by 3–5 °C in the composites with modified celluloses (Table 4). The shift of the T_g_ to a higher temperature was due to the restriction of polymer chain movements. This may be caused by the interactions between PHB and the modified celluloses [19,37], which are also indicated by the lower intensity of the tan δ peak in composites compared to PHB, except for PHB/MC-SIV-MA (Figure 8). The increased damping in the composite with MC-SIV-MA was a result of the higher content of amorphous phase in PHB/MC-SIV-MA, with 42.9% crystallinity instead of 50–51% for the other composites (Table 3). In addition, the breadth of the tan δ peak was larger for PHB/MC-SIMA-MA because of the difference in PHB chains’ mobility; the movements of the PHB chains close to the modified cellulose fibers being much more restrained than those of the farthest ones [14]. Indeed, the methacrylate groups and polymethacrylate grafts on modified celluloses show good interactions with the PHB matrix thanks to their compatibility [22].

Microfibrillated celluloses had a small influence on the storage modulus of PHB, except for MC-SIMA-MA, which led to higher E’ values on the whole tested temperature range (Table 4). Thus, an increase in the storage modulus by up to 23% was noticed in PHB/MC-SIMA-MA compared to the PHB reference. The reinforcing efficiency of MC and modified celluloses in PHB composites was assessed by the effectiveness coefficient (C), which is the ratio of the storage modulus values in the glassy and rubbery regions for the composite reported inthe similar ratio for the matrix [11]. The values of E’ at −25 °C and 100 °C were used for the storage modulus in the glassy and rubbery regions. The lowest C values, corresponding to the highest reinforcing effectiveness of cellulose fibers, were obtained for the PHB/MC-SIMA-MA composites (Table 4). Therefore, the treatment of MC with SIMA and MA improved the compatibility of cellulose fibers with the PHB matrix and increased the mechanical properties. In contrast, the treatment of MC with SIV and MA led to an opposite effect. Indeed, the lowest E’ values of almost the entire temperature range were obtained for PHB/MC-SIV-MA. The poor polymerization of methacrylic acid on the SIV-modified MC, as demonstrated by FTIR, may explain this behavior.

#### 3.3.5. Tensile Properties of the Composites

**Figure 9:**
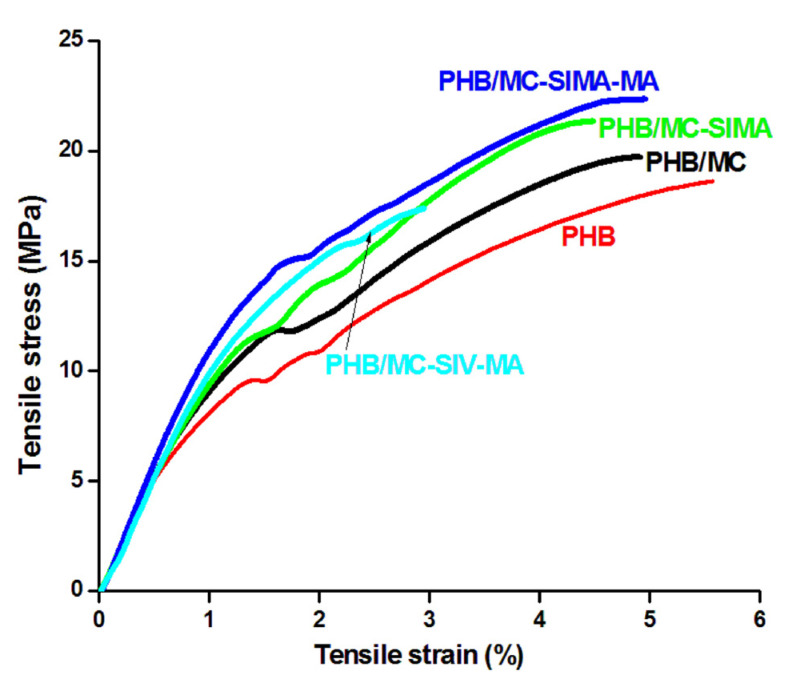
Representative stress–strain curves of PHB and composites.

The mechanical properties of PHB and composites, elongation at break, tensile strength at break (σ), and Young’s modulus (M) are presented in Table 5, and the representative stress–strain curves in Figure 9. Without any surface treatment, MC had a poor effect on the mechanical properties of PHB;σ increased by 6%, which is in the limit of the experimental error, and M by 10%. A higher increase inthe tensile strength was noticed in the composites with modified celluloses, in PHB/MC-SIMA by 13% and in PHB/MC-SIMA-MA by 18%. In the second composite, the Young’s modulus increased byalmost 30%. The increase inthe mechanical properties was higher than that reported for a PHBV/2.5% nanofibrillated cellulose composite [34]. Thus, the reinforcing effect observed in the composite containing MC-SIMA-MA proved the effectiveness of this surface treatment of cellulose fibers, which increased the interfacial bonding between PHB and cellulose. Indeed, the polymerization reaction of methacrylic acid on the SIMA-modified cellulose resulted in a compatibilization with the PHB matrix. A drastic decrease inall mechanical properties was observed in PHB/MC-SIV-MA. The opposite effect forMC-SIV-MA may be due to the ineffective treatment of cellulose when vinyl silane groups were involved, as also demonstrated by FTIR. The degree of crystallinity also has a strong influence on the mechanical properties. The increase incrystallinity, determined by the nucleating effect of cellulose fibers, was similar in all composites with the exception of PHB/MC-SIV-MA (Table 3). In this case, X_c_ decreased bymore than 10%, and this can be considered as an important cause of the drop in the mechanical properties andof inefficient treatment.

**Figure 10:**
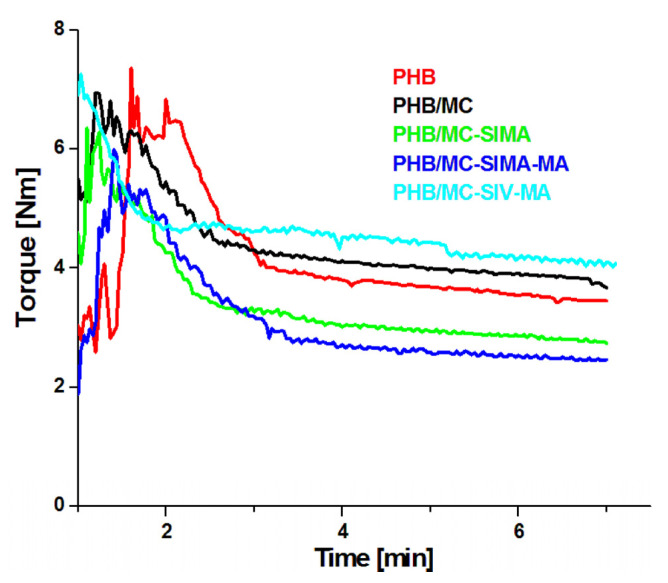
Torque vs. time diagrams recorded during the melt processing of PHB and composites.

Looking at the stress–strain curves of composites (Figure 9), one may observe that the reinforcing effect of MC-SIMA-MA and MC-SIMA was not followed by a strong decrease inelongation at break, as is the case for MC-SIV-MA and in the literature [34]. This behavior may result from a plasticizing effect of SIMA and polymethacrylic acid grafts. For verifying this hypothesis, the variation in time of the torque during the melt processing of the samples was analyzed (Figure 10).

The two composites, PHB/MC-SIMA and PHB/MC-SIMA-MA, showed lower viscosity than PHB and PHB/MC. Therefore, these treatments for the surface modification of MC not only have a compatibilizing effect in PHB, but also a plasticizing one. This is an important finding because the addition of fillers in PHB generally increases its brittleness, which is already large and deteriorates its processability. Therefore, the double role of SIMA-MA treatment, as both compatibilizer and plasticizer, could better solve the issues related to PHB application. Moreover, the overlap of the plasticizing effect of modified celluloses with the reinforcing effect can, to a certain extent, diminish the increase in tensile strength and modulus, leading to much more balanced stiffness–toughness properties.

#### 3.3.6. Morphological Investigation of Composites

**Figure 11:**
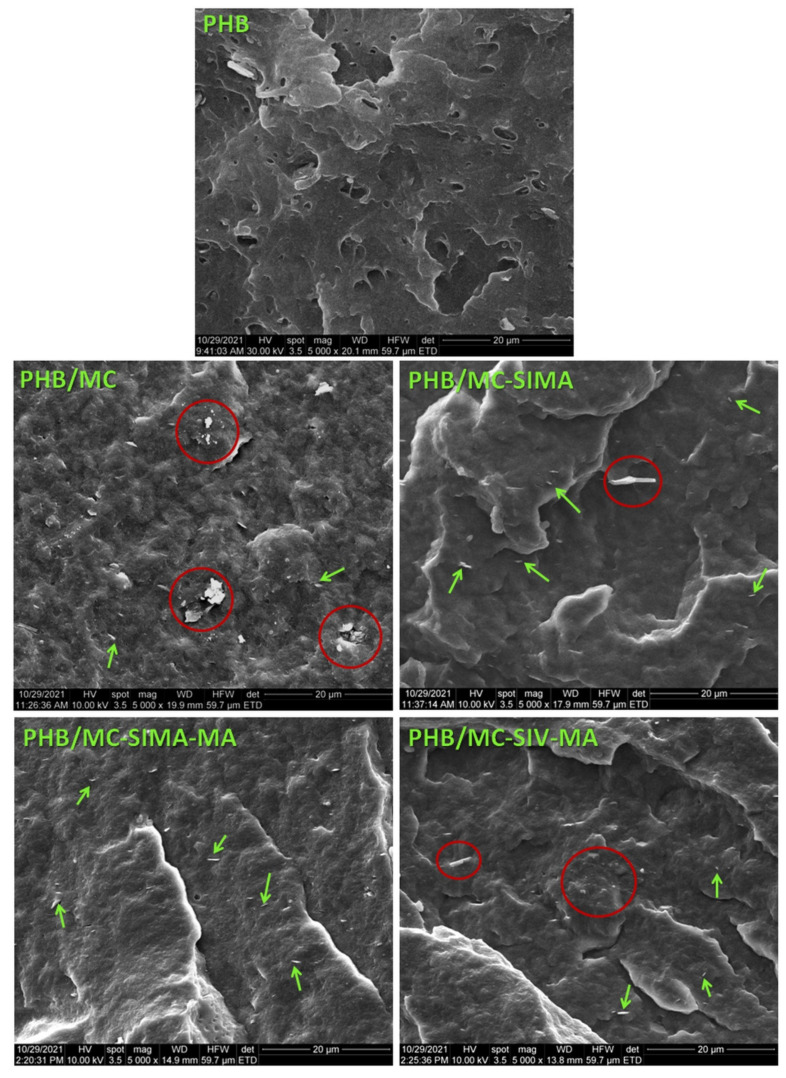
SEM images of PHB and composites frozen in liquid nitrogen and fractured.

The SEM images in the fracture of PHB and composite plates are shown in Figure 11. In fracture, PHB shows pores of different sizes and several impurities. These are probably additives with different purposes than commercial PHB. The PHB composite with untreated MC shows many agglomerations of fibers, encircled with a red line in Figure 11 (PHB/MC).

In the SEM images of the composites with modified celluloses, the dispersion of cellulose fibers was much better, especially in PHB/MC-SIMA-MA. Many individual fibers, marked with green arrows, can be seen in these images. Their thickness was less than 100 nm. It should be mentioned that in the composites with modified celluloses the pores appeared only scarcely. The morphological features of the composites with modified celluloses support their improved mechanical behavior.

## 4. Conclusions

This study was the first attempt to use a different strategy for the chemical modification of the surface of cellulose by firstly using silanes to create active sites, followed by the graft polymerization of methacrylic acid. FTIR spectroscopy confirmed the high efficiency of the SIMA and SIMA-MA treatments and the low efficiency of the SIV-MA treatment. The good adhesion between SIMA-modified celluloses and PHB and their good dispersion, highlighted by SEM, led to a significant improvement in the tensile strength and modulus in these composites; an opposite effect was noticed for PHB/MC-SIV-MA. Furthermore, the effectiveness coefficient, calculated from the storage modulus data, highlighted the reinforcing effect of MC-SIMA-MA and the unfavorable effect of MC-SIV-MA. The beneficial effect of SIMA-modified celluloses in PHB was also supported by the increased crystallinity and the smaller spherulites formed in this composite, as observed from the differential scanning calorimetry and polarized optical microscopy analyses. In addition, SIMA and SIMA-MA treatments had a dual role in achieving both compatibilization and plasticization. Therefore, this new method to modify cellulose fibers proved to be facile and effective when considering the improvements in the thermal and overall mechanical properties of the PHB matrix.

## Figures and Tables

**Table 1:** Thermogravimetric data for the modified celluloses.

Sample	MC	MC-SIMA	MC-SIMA-MA	MC-SIV-MA
T_5%_, °C	270.7	305.1	290.6	284.5
T_max_, °C	348.7	356.5	335.5	353.2
WL_200°C_, %	3.8	1.4	2.7	2.5
Residue at 700 °C, %	1.4	24.4	12.0	6.8

**Table 2:** TGA data for the PHB composites with differently modified celluloses.

Composites	PHB	PHB/MC	PHB/MC-SIMA	PHB/MC-SIMA-MA	PHB/MC-SIV-MA
T_5%_, °C	246.1	245.5	242.4	244.5	246.8
T_max_, °C	292.4	290.9	287.9	290	288.6
Residue at 700 °C, %	1.3	1.3	1.7	2.0	1.3

**Table 3:** DSC data for the composites.

Composites	First Heating		Cooling		Second Heating		X_c_ (%)
	T_m1_(°C)	ΔH_m1_ (J/g)	T_c_ (°C)	ΔH_c_ (J/g)	T_m2_ (°C)	ΔH_m2_ (J/g)	
PHB	158.4/168.0	67.3	111.3	62.5	160.9	69.6	47.7
PHB/MC	159.0/168.2	67.3	112.3	64.2	160.9	72.3	50.3
PHB/MC-SIMA	158.9/168.2	69.1	112.8	64.9	160.2	73.6	51.4
PHB/MC-SIMA-MA	159.4/168.5	67.8	112.8	64.2	160.7	72.5	50.7
PHB/MC-SIV-MA	160.1/168.2	56.2	113.0	57.2	161.0	61.5	42.9

**Table 4:** Storage modulus (E’) of the composites at different temperatures, glass transition temperature (T_g_) determined from tan δ vs. temperature curve and tan δ value at T_g_.

Composites	PHB	PHB/MC	PHB/MC-SIMA	PHB/MC-SIMA-MA	PHB/MC-SIV-MA
E’_−25°C_, MPa	5569	5484	5488	5674	5460
E’_0°C_, MPa	3817	3783	3741	4041	3722
E’_25°C_, MPa	2660	2659	2671	2844	2486
E’_50°C_, MPa	2116	2099	2087	2242	1880
E’_100°C_, MPa	844	938	943	1040	863
T_g_, °C	6.8	6.9	9.3	10.9	12.2
tan δ	0.060	0.066	0.059	0.059	0.067
C	-	0.886	0.882	0.827	0.959

**Table 5:** Tensile properties data of composites.

Composites	PHB	PHB/MC	PHB/MC-SIMA	PHB/MC-SIMA-MA	PHB/MC-SIV-MA
Elongation at break, %	5.3 ± 0.6	4.9 ± 0.4	4.5 ± 0.6	5.0 ± 0.2	3.0 ± 0.5
Tensile strength at break, MPa	18.7 ± 1.9	19.8 ± 1.6	21.1 ± 0.7	22.0 ± 0.3	17.4 ± 2.0
Young’s modulus, MPa	868 ± 58	954 ± 42	946 ± 61	1116 ± 12	966 ± 17

## Data Availability

The data presented in this study are available on request from the corresponding author.
